# Cell-free synthetic biology for *in vitro* prototype engineering

**DOI:** 10.1042/BST20170011

**Published:** 2017-06-15

**Authors:** Simon J. Moore, James T. MacDonald, Paul S. Freemont

**Affiliations:** Department of Medicine, Centre for Synthetic Biology and Innovation, South Kensington Campus, London, U.K.

**Keywords:** cell free, gene expression, modelling, prototyping, synthetic biology

## Abstract

Cell-free transcription–translation is an expanding field in synthetic biology as a rapid prototyping platform for blueprinting the design of synthetic biological devices. Exemplar efforts include translation of prototype designs into medical test kits for on-site identification of viruses (Zika and Ebola), while gene circuit cascades can be tested, debugged and re-designed within rapid turnover times. Coupled with mathematical modelling, this discipline lends itself towards the precision engineering of new synthetic life. The next stages of cell-free look set to unlock new microbial hosts that remain slow to engineer and unsuited to rapid iterative design cycles. It is hoped that the development of such systems will provide new tools to aid the transition from cell-free prototype designs to functioning synthetic genetic circuits and engineered natural product pathways in living cells.

## Introduction

Cell-free systems represent a historically important component of the founding of the field of biochemistry. Ever since the pioneering efforts of the Nobel laureate Eduard Buchner (Nobel Prize in Chemistry in 1907) and his discovery of fermentation in yeast cell extracts [1], cell-free systems have been repurposed towards the further understanding of biological processes. Indeed, arguably one of the most notable biological discoveries of the 20th century was the unravelling of the genetic code by Nirenberg and colleagues [2–4], which was underpinned by the use of *Escherichia coli* cell extracts to study coupled transcription–translation (TX–TL). Together with Har Khorana and Robert Holley, this resulted in a shared Novel Prize in Physiology or Medicine in 1968. On this theme, the efforts of Alfred Goldberg led to the unveiling of an ATP-dependent mechanism for protein degradation by ubiquitin in a mammalian cell-free system [5].

### Cell-free synthetic biology

Today, with the rise of synthetic biology and the design and construction of synthetic life [6], cell-free systems have yet again found a niche towards the understanding of biological networks and biosynthetic pathways [7,8]. Indeed, by isolating the cellular components of core metabolism and the TX–TL network within a test tube, this allows the synthetic biologist to study systems without the regulatory constraints and limitations of a dividing, evolving or adapting living cell. This mini-review summarises the efforts of recent cell-free synthetic biology research and the opportunities it provides for the future.

**Figure 1.**
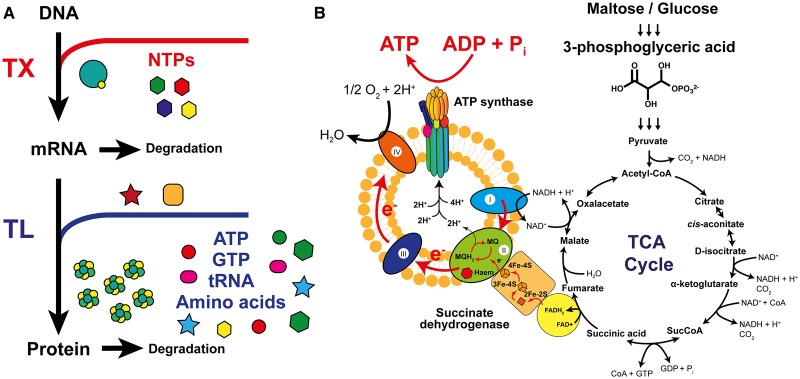
Summary of cell-free TX–TL. (**A**) TX and TL process and requirements of NTPs and substrates (ATP, GTP, tRNA and amino acids). (**B**) Energy regeneration cycle for central metabolism. ATP is synthesised through the formation of inverted vesicles, which spontaneously form during cell disruption [37]. Abbreviations: MQ, menaquinone; MQH_2_, reduced menaquinone.

Cell-free coupled TX–TL utilises the core machinery of RNA polymerase holoenzyme, the translation apparatus (ribosomes, tRNA synthetases and translation factors) and energy regeneration enzymes to amplify a set of DNA instructions into the target protein(s) of choice (Figure 1A). Therefore, the study of cell-free presents an enticing opportunity to the synthetic biologist to design and engineer living systems from the bottom-up as prototype designs. Exemplar demonstrations of cell-free synthetic biology include their use as biomolecular ‘breadboards’ [9,10], healthcare biosensors [11,12] and enzyme cascades [13–16]. Coupled with the aid of computational design approaches [10,17–19], these early developments in cell-free synthetic biology will endeavour to aid the engineering of more complex systems. We shall now summarise the cell-free platforms available, with a specific focus to its use in prototyping genetic circuits.

### *E. coli* cell-free — purified recombinant elements or crude cell extract?

The choice of a well-characterised cell-free system almost entirely resides with *E. coli* platforms, which are based on either a crude cell extract [20–23] or a system of purified recombinant elements (PURE) [24,25]. A vital area of importance to cell-free systems is the process of energy regeneration, which represents the major cost factor and limitation for both the PURE and cell extract-based routes. First, transcription requires nucleotide triphosphates (NTPs — ATP, UTP, GTP and CTP), with each mRNA transcript utilised multiple times for protein synthesis [26]. Protein translation is the major energy cost factor and requires two ATP equivalents for tRNA aminoacylation and two GTP equivalents per peptide bond formed [27]. In addition, a single GTP equivalent is required for each of the initiation and termination steps. Therefore, a small sized 25 kDa protein costs ∼35–44 mM of ATP to synthesise 1 mg/ml under batch synthesis [27].

First, in respect to the PURE system [25], this includes the purified components (108 in total) of the entire *E. coli* translation machinery including ribosomes, 22 tRNA synthetases, initiation factors, elongation, release and termination factors, which when combined with T7 RNA polymerase, tRNA, energy regeneration enzymes, substrates (amino acids and creatine phosphate) and synthetic DNA instructions, this reconstitutes the entire TX–TL network within a test tube. This rather remarkable engineering feat is commercially available as the PURExpress® kit (New England Biolabs). While the high cost of the system prohibits scaled-up applications, a variety of cell-free researchers utilise the PURExpress® system to study the dynamics and kinetics of TX–TL [24,28–32]. The major advantage of the PURE system is it's high efficiency due to an absence of competing side reactions such as non-specific phosphatases [24], which rapidly degrade the energy source.

In contrast, a crude cell extract provides an inexpensive route to protein synthesis. In addition, unlike the PURE system, reactions are scalable into high-volume fermentation conditions [33,34]. However, with the presence of other primary and secondary pathway enzymes (phosphatases and amino acid biosynthesis), this leads to undesirable side reactions during catabolism of the starting energy source. Importantly, based on improvements in energy regeneration schemes by the groups of Swartz [27,35–37], Jewett [38,39] and Noireaux [40–42], powerful cell extract-based batch systems can now reach recombinant protein yields of up to 2.34 mg/ml [33,40], while extended steady-state synthesis can be achieved through the use of a semi-permeable dialysis membrane device, thus elevating protein yields up to 6 mg/ml [40]. In addition, inexpensive energy sources such as glucose [27], glutamate [33], maltose/maltodextrin [41] and succinate [43] can be utilised to reduce the cost of energy regeneration in cell-free systems (Figure 1B). To this end, various cell extract protocols have been developed and are based on the harvesting of cells at exponential phase, when typically intracellular translation is at its peak. Standardised protocols involve washing the cells, mechanical lysis [38] and activation of the extract through a run-off reaction, a process believed to degrade endogenous mRNA transcripts and genomic DNA that can reduce cell-free translation efficiency [23]. Additional dialysis can also remove inhibitory small molecules, but the requirement of this varies between *E. coli* strains and user preference [38].

### Cell-free prototyping

**Figure 2.**
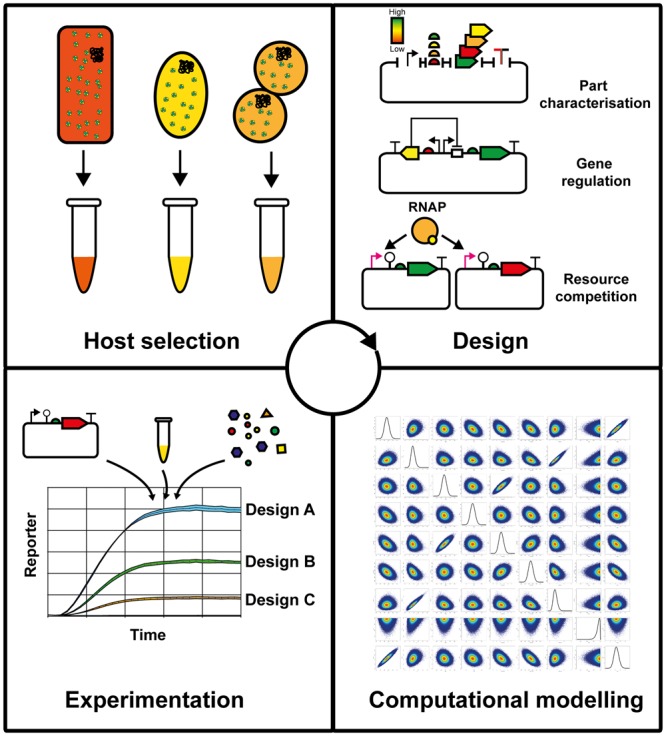
Prototyping cell-free TX–TL systems. A workflow for the prototyping of new microbial platforms, coupled with DNA libraries, testing and computational modelling.

Cell-free TX–TL provides the ability to study gene expression in isolation with the timescale from DNA to experimental results taking a few hours [10,44,45], whereas depending on the host chassis, an *in vivo* based approach can take several days to weeks. Thus, cell-free provides a prototyping approach (Figure 2) for rapid cycling between circuit experimental design and debugging [9]. To enable cell-free prototyping, fluorescence tags that monitor both mRNA and protein synthesis can be studied in real time [29,40,46,47], thus providing dual microscale quantitative data of the TX–TL cascade that can be difficult to achieve within a living cell. In addition, the starting concentration of the substrates and relative enzyme stoichiometry can be determined [40], thus aiding system identification and mathematical modelling of the chemical reaction dynamics [9,17,18]. These models can be used to inform future circuit designs as part of an iterative design process. *In vivo*, the cellular components are constantly being diluted by cell growth and division as well as being synthesised. In contrast, batch cell-free reactions are closed systems starting with a limited set of initial resources. These differences make direct comparisons between *in vivo* and cell-free reaction dynamics of complex multi-promoter circuits difficult. One method to combine the rapid prototyping benefits of cell-free while emulating the conditions found in living cells is to use microfluidic devices to allow the continuous dilution and replenishment of the reaction substrates. This method was used to design three- and five-node ring oscillators in cell-free, based on the utilisation of PCR templates to test initial prototypes, before a model-inspired design-build-test cycle led to circuit designs that were also found to function in cells [48]. On this theme, cell-free provides a dynamic biochemical system that can be accurately described by mathematical modelling [9].

In another context, cell-free prototyping can also be useful towards the design of synthetic cells. At the systems level, central to this effort is the further understanding of cellular compartmentalisation. Owing to its difficulty, especially at the structural level, a perhaps understudied area of biology is the dynamics of protein folding in the lipid membrane bilayer. Cell-free uniquely provides an opportunity to study the folding of membrane proteins [49], while in synthetic microfluidic-based liposomes, enzymes and substrates can be transported from one cell to another, demonstrating a simple recreation of membrane trafficking [50]. Towards complexity, cell-free systems have also begun to be implemented for the assembly of large protein complexes. The bacteriophage represents a simple life form with a package of genes within a protein shell, which is released upon invasion of a cell to hijack the hosts TX–TL apparatus. A classically studied system is the T7 bacteriophage [51] that infects *E. coli*. Through cell-free, it has now been shown possible that the 40 kb dsDNA genome of the T7 bacteriophage, which constitutes 57 genes, can be reconstituted *in vitro* to demonstrate the assembly of a natural protein compartment [52]. This is also expandable to other bacteriophage systems [40]. Moving aside beyond biological compartmentalisation, cell-free is also portable to the interface of nanotechnology for studying gene expression and synthesising protein nanotube on biochips [53]. Together these examples of compartmentalisation demonstrate an extra level of complexity in cell-free systems for prototype designs, which may aid in the design of new synthetic cells in the future.

### Non-model cell-free platforms

Viewed from a different perspective, synthetic biology has begun to examine the prospects of engineering non-model microbial hosts [54] that can provide unique advantages for biotechnological application, such as rapid growth with inexpensive substrates, growth in extreme conditions or unique enzyme machinery, which in some cases can only accessed within non-standard microbial hosts. However, the greatest disadvantage of such cultivatable microbes is a combination of one or more of the following traits, such as a general lack of characterised gene expression tools, poor genetic tractability or insufficient knowledge towards the microbe metabolism. While cell-free cannot directly address genetic competence, it could provide a starting point to understanding the host's inherent TX–TL kinetics, genetic tools and enzymology, without the time limitations associated with direct engineering of the host. Noticeably, the methodology for cell-free extract preparation [42,55] has shown universal application to a variety of microbial cell-free platforms such as *Saccharomyces cerevisiae* [56,57], *Streptomyces* spp. [58–61] and *Bacillus* spp. [43,62]. Such interest in the use and application of alternative cell-free systems as a prototyping device is likely to grow; however, the cooperative development of synthetic biology tools with translational application into live cells may provide the greatest opportunity to access the design space of traditionally difficult to engineer microbes. In particular reference to the *Streptomyces* family, the high G + C (%) soil bacteria, it has long been appreciated that these hosts provide unique and well-characterised platform for the assembly of a rich repertoire of natural products [63]. For *Streptomyces* cell-free, the recently developed high-yielding *Streptomyces lividans* and *Streptomyces venezuelae* host platforms [58,61] can potentially provide an opportunity to access high G + C (%) enzymes from secondary metabolism directly within a test tube for combinatorial biosynthesis. With further advances in efficiency and yield, *Streptomyces* cell-free could be utilised for incorporating non-natural or potentially toxic substrates into natural products, towards expanding the chemical space of biosynthesis. A proof of concept of how cell-free can be utilised to incorporate non-natural amino acids into protein backbones was demonstrated for GFP synthesis in *E. coli* cell-free [64]. While this technology is in its infancy, it is also possible to engineer this in living cells in high yield [65], which has been made available through the multiplex automated genome engineering (MAGE) technology [66]. However, this methodology is currently only accessible to specially engineered strains of *E. coli*. Thus, with further developments, cell-free potentially provides a potentially fast route to prototype and engineer the application of novel chemistry to natural product biosynthesis [67].

### Conclusions

The rise of cell-free systems from its historical links in foundational biochemistry has provided a platform to this expanding field in synthetic biology. Perhaps, the greatest challenge of cell-free studies is to establish and define the boundaries and limitations of mimicking cellular biology within cell-free systems. One understudied area is the impact of molecular crowding on enzyme velocities [68,69] and spatial organisation [70], which can only be artificially controlled in cell-free reactions. In fact, cell-free systems in essence are reminiscent of primordial biology [71], whereby enzymes (or ribozymes) and chemicals once freely tumbled without the restrictions of biological compartmentalisation and the regulatory control of the genome. With the growing interest in the design of a minimal synthetic cell [72–74], cell-free systems can provide a base towards the design of synthetic life from individual components. We anticipate that the prototyping and modelling of gene expression and enzyme machinery from understudied arcane microbes will provide important new tools for the cell-free synthetic biologist's disposal.

## Abbreviations

NTPs: nucleotide triphosphates
PURE: purified recombinant elements
TX-TL: transcription–translation

## References

[1] Jaenicke L. (2007) Centenary of the award of a Nobel Prize to Eduard Buchner, the father of biochemistry in a test tube and thus of experimental molecular bioscience. Angew. Chem. Int. Ed. Engl. 46, 6776–6782 doi:10.1002/anie.200700390 17600804

[2] Matthaei J.H. and Nirenberg M.W. (1961) Characteristics and stabilization of DNAase-sensitive protein synthesis in *E. coli* extracts. Proc. Natl Acad. Sci. U.S.A. 47, 1580–1588 doi:10.1073/pnas.47.10.1580 14471391 PMC223177

[3] Matthaei J.H., Jones O.W., Martin R.G. and Nirenberg M.W. (1962) Characteristics and composition of RNA coding units. Proc. Natl Acad. Sci. U.S.A. 48, 666–677 doi:10.1073/pnas.48.4.666 14471390 PMC220831

[4] Nirenberg M.W. and Matthaei J.H. (1961) The dependence of cell-free protein synthesis in *E. coli* upon naturally occurring or synthetic polyribonucleotides. Proc. Natl Acad. Sci. U.S.A. 47, 1588–1602 doi:10.1073/pnas.47.10.1588 14479932 PMC223178

[5] Etlinger J.D. and Goldberg A.L. (1977) A soluble ATP-dependent proteolytic system responsible for the degradation of abnormal proteins in reticulocytes. Proc. Natl Acad. Sci. U.S.A. 74, 54–58 doi:10.1073/pnas.74.1.54 264694 PMC393195

[6] Nielsen A.A.K., Der B.S., Shin J., Vaidyanathan P., Paralanov V., Strychalski E.A. et al. (2016) Genetic circuit design automation. Science 352, aac7341 doi:10.1126/science.aac7341 27034378

[7] Hodgman C.E. and Jewett M.C. (2012) Cell-free synthetic biology: thinking outside the cell. Metab. Eng. 14, 261–269 doi:10.1016/j.ymben.2011.09.002 21946161 PMC3322310

[8] Hockenberry A.J. and Jewett M.C. (2012) Synthetic *in vitro* circuits. Curr. Opin. Chem. Biol. 16, 253–259 doi:10.1016/j.cbpa.2012.05.179 22676890 PMC3424401

[9] Siegal-Gaskins D., Tuza Z.A., Kim J., Noireaux V. and Murray R.M. (2014) Gene circuit performance characterization and resource usage in a cell-free ‘breadboard’. ACS Synth. Biol. 3, 416–425 doi:10.1021/sb400203p 24670245

[10] Sun Z.Z., Yeung E., Hayes C.A., Noireaux V. and Murray R.M. (2014) Linear DNA for rapid prototyping of synthetic biological circuits in an *Escherichia coli* based TX–TL cell-free system. ACS Synth. Biol. 3, 387–397 doi:10.1021/sb400131a 24303785

[11] Pardee K., Green A.A., Takahashi M.K., Braff D., Lambert G., Lee J.W. et al. (2016) Rapid, low-cost detection of Zika virus using programmable biomolecular components. Cell 165, 1255–1266 doi:10.1016/j.cell.2016.04.059 27160350

[12] Pardee K., Green A.A., Ferrante T., Cameron D.E., Daleykeyser A., Yin P. et al. (2014) Paper-based synthetic gene networks. Cell 159, 940–954 doi:10.1016/j.cell.2014.10.004 25417167 PMC4243060

[13] Bujara M., Schümperli M., Billerbeck S., Heinemann M. and Panke S. (2010) Exploiting cell-free systems: implementation and debugging of a system of biotransformations. Biotechnol. Bioeng. 106, 376–389 doi:10.1002/bit.22666 20091765

[14] You C. and Zhang Y.-H.P. (2014) Annexation of a high-activity enzyme in a synthetic three-enzyme complex greatly decreases the degree of substrate channeling. ACS Synth. Biol. 3, 380–386 doi:10.1021/sb4000993 24283966

[15] Dudley Q.M., Karim A.S. and Jewett M.C. (2015) Cell-free metabolic engineering: biomanufacturing beyond the cell. Biotechnol. J. 10, 69–82 doi:10.1002/biot.201400330 25319678 PMC4314355

[16] Karim A.S. and Jewett M.C. (2016) A cell-free framework for rapid biosynthetic pathway prototyping and enzyme discovery. Metab. Eng. 36, 116–126 doi:10.1016/j.ymben.2016.03.002 26996382

[17] Karzbrun E., Shin J., Bar-Ziv R.H. and Noireaux V. (2011) Coarse-grained dynamics of protein synthesis in a cell-free system. Phys. Rev. Lett. 106, 048104 doi:10.1103/PhysRevLett.106.048104 21405367

[18] Tuza Z.A., Singhal V., Kim J. and Murray R.M. (2013) An in silico modeling toolbox for rapid prototyping of circuits in a biomolecular ‘breadboard’ system. 52nd IEEE Conference on Decision and Control, Firenze, pp. 1404–1410. doi:10.1109/CDC.2013.6760079.

[19] Takahashi M.K., Chappell J., Hayes C.A., Sun Z.Z., Kim J., Singhal V. et al. (2015) Rapidly characterizing the fast dynamics of RNA genetic circuitry with cell-free transcription–translation (TX–TL) systems. ACS Synth. Biol. 4, 503–515 doi:10.1021/sb400206c 24621257 PMC4487224

[20] Schwarz D., Junge F., Durst F., Frölich N., Schneider B., Reckel S. et al. (2007) Preparative scale expression of membrane proteins in *Escherichia coli*-based continuous exchange cell-free systems. Nat. Protoc. 2, 2945–2957 doi:10.1038/nprot.2007.426 18007631

[21] Kim D.-M., Kigawa T., Choi C.-Y. and Yokoyama S. (1996) A highly efficient cell-free protein synthesis system from *Escherichia coli*. Eur. J. Biochem. 239, 881–886 doi:10.1111/j.1432-1033.1996.0881u.x 8774739

[22] Kigawa T., Yabuki T., Matsuda N., Matsuda T., Nakajima R., Tanaka A. et al. (2004) Preparation of *Escherichia coli* cell extract for highly productive cell-free protein expression. J. Struct. Funct. Genomics 5, 63–68 doi:10.1023/B:JSFG.0000029204.57846.7d 15263844

[23] Liu D.V., Zawada J.F. and Swartz J.R. (2005) Streamlining *Escherichia coli* S30 extract preparation for economical cell-free protein synthesis. Biotechnol. Prog. 21, 460–465 doi:10.1021/bp049789y 15801786

[24] Kuruma Y. and Ueda T. (2015) The PURE system for the cell-free synthesis of membrane proteins. Nat. Protoc. 10, 1328–1344 doi:10.1038/nprot.2015.082 26270393

[25] Shimizu Y., Inoue A., Tomari Y., Suzuki T., Yokogawa T., Nishikawa K. et al. (2001) Cell-free translation reconstituted with purified components. Nat. Biotechnol. 19, 751–755 doi:10.1038/90802 11479568

[26] Kim D.-M. and Swartz J.R. (2001) Regeneration of adenosine triphosphate from glycolytic intermediates for cell-free protein synthesis. Biotechnol. Bioeng. 74, 309–316 doi:10.1002/bit.1121 11410855

[27] Calhoun K.A. and Swartz J.R. (2007) Energy systems for ATP regeneration in cell-free protein synthesis reactions. Methods Mol. Biol. 375, 3–17 PMID:17634594 10.1007/978-1-59745-388-2_1

[28] Geertz M., Shore D. and Maerkl S.J. (2012) Massively parallel measurements of molecular interaction kinetics on a microfluidic platform. Proc. Natl Acad. Sci. U.S.A. 109, 16540–16545 doi:10.1073/pnas.1206011109 23012409 PMC3478601

[29] Niederholtmeyer H., Stepanova V. and Maerkl S.J. (2013) Implementation of cell-free biological networks at steady state. Proc. Natl Acad. Sci. U.S.A. 110, 15985–15990 doi:10.1073/pnas.1311166110 24043836 PMC3791785

[30] Chizzolini F., Forlin M., Yeh Martín N., Berloffa G., Cecchi D. and Mansy S.S. (2017) Cell-free translation is more variable than transcription. ACS Synth. Biol. doi:10.1021/acssynbio.6b00250 28100049

[31] de Maddalena L.L., Niederholtmeyer H., Turtola M., Swank Z.N., Belogurov G.A. and Maerkl S.J. (2016) Grea and greB enhance expression of *Escherichia coli* RNA polymerase promoters in a reconstituted transcription–translation system. ACS Synth. Biol. 5, 929–935 doi:10.1021/acssynbio.6b00017 27186988

[32] Karig D.K., Iyer S., Simpson M.L. and Doktycz M.J. (2012) Expression optimization and synthetic gene networks in cell-free systems. Nucleic Acids Res. 40, 3763–3774 doi:10.1093/nar/gkr1191 22180537 PMC3333853

[33] Cai Q., Hanson J.A., Steiner A.R., Tran C., Masikat M.R., Chen R. et al. (2015) A simplified and robust protocol for immunoglobulin expression in *Escherichia coli* cell-free protein synthesis systems. Biotechnol. Prog. 31, 823–831 doi:10.1002/btpr.2082 25826247 PMC5029582

[34] Zawada J.F., Yin G., Steiner A.R., Yang J., Naresh A., Roy S.M. et al. (2011) Microscale to manufacturing scale-up of cell-free cytokine production-a new approach for shortening protein production development timelines. Biotechnol. Bioeng. 108, 1570–1578 doi:10.1002/bit.23103 21337337 PMC3128707

[35] Jewett M.C. and Swartz J.R. (2004) Mimicking the *Escherichia coli* cytoplasmic environment activates long-lived and efficient cell-free protein synthesis. Biotechnol. Bioeng. 86, 19–26 doi:10.1002/bit.20026 15007837

[36] Yang W.C., Patel K.G., Wong H.E. and Swartz J.R. (2012) Simplifying and streamlining *Escherichia coli*-based cell-free protein synthesis. Biotechnol. Prog. 28, 413–420 doi:10.1002/btpr.1509 22275217

[37] Jewett M.C., Calhoun K.A., Voloshin A., Wuu J.J. and Swartz J.R. (2008) An integrated cell-free metabolic platform for protein production and synthetic biology. Mol. Syst. Biol. 4, 220 doi:10.1038/msb.2008.57 18854819 PMC2583083

[38] Kwon Y.-C. and Jewett M.C. (2015) High-throughput preparation methods of crude extract for robust cell-free protein synthesis. Sci. Rep. 5, 8663 doi:10.1038/srep08663 25727242 PMC4345344

[39] Harris D.C. and Jewett M.C. (2012) Cell-free biology: exploiting the interface between synthetic biology and synthetic chemistry. Curr. Opin. Biotechnol. 23, 672–678 doi:10.1016/j.copbio.2012.02.002 22483202 PMC4038125

[40] Garamella J., Marshall R., Rustad M. and Noireaux V. (2016) The all *E. coli* TX–TL Toolbox 2.0: a platform for cell-free synthetic biology. ACS Synth. Biol. 5, 344–355 doi:10.1021/acssynbio.5b00296 26818434

[41] Caschera F. and Noireaux V. (2014) Synthesis of 2.3 mg/ml of protein with an all *Escherichia coli* cell-free transcription–translation system. Biochimie 99, 162–168 doi:10.1016/j.biochi.2013.11.025 24326247

[42] Sun Z.Z., Hayes C.A., Shin J., Caschera F., Murray R.M. and Noireaux V. (2013) Protocols for implementing an *Escherichia coli* based TX–TL cell-free expression system for synthetic biology. J. Vis. Exp. 50762, e50762 doi:10.3791/50762 PMC3960857 24084388

[43] Moore S.J., MacDonald J.T., Weinecke S., Kylilis N., Polizzi K.M., Biedendieck R. et al. (2016) Prototyping of *Bacillus megaterium* genetic elements through automated cell-free characterization and Bayesian modelling. bioRxiv doi:10.1101/071100

[44] Moore S.J., Lai H.-E., Kelwick R.J.R., Chee S.M., Bell D., Polizzi K.M. et al. (2016) EcoFlex — a multifunctional MoClo Kit for *E. coli* synthetic biology. ACS Synth. Biol. 5, 1059–1069 doi:10.1021/acssynbio.6b00031 27096716

[45] Chappell J., Jensen K. and Freemont P.S. (2013) Validation of an entirely in vitro approach for rapid prototyping of DNA regulatory elements for synthetic biology. Nucleic Acids Res. 41, 3471–3481 doi:10.1093/nar/gkt052 23371936 PMC3597704

[46] Niederholtmeyer H., Xu L. and Maerkl S.J. (2013) Real-time mRNA measurement during an *in vitro* transcription and translation reaction using binary probes. ACS Synth. Biol. 2, 411–417 doi:10.1021/sb300104f 23654250

[47] Kim J., Khetarpal I., Sen S. and Murray R.M. (2014) Synthetic circuit for exact adaptation and fold-change detection. Nucleic Acids Res. 42, 6078–6089 doi:10.1093/nar/gku233 24728988 PMC4027175

[48] Niederholtmeyer H., Sun Z.Z., Hori Y., Yeung E., Verpoorte A., Richard M. et al. (2015) Rapid cell-free forward engineering of novel genetic ring oscillators. eLife 4, e09771 doi:10.7554/eLife.09771 26430766 PMC4714972

[49] Findlay H.E., Harris N.J. and Booth P.J. (2016) In vitro synthesis of a major facilitator transporter for specific active transport across droplet interface bilayers. Sci. Rep. 6, 39349 doi:10.1038/srep39349 27996025 PMC5172200

[50] Elani Y., Law R.V. and Ces O. (2014) Vesicle-based artificial cells as chemical microreactors with spatially segregated reaction pathways. Nat. Commun. 5, 5305 doi:10.1038/ncomms6305 25351716

[51] Chamberlin M., McGrath J. and Waskell L. (1970) New RNA polymerase from *Escherichia coli* infected with bacteriophage T7. Nature 228, 227–231 doi:10.1038/228227a0 4920917

[52] Shin J., Jardine P. and Noireaux V. (2012) Genome replication, synthesis, and assembly of the bacteriophage T7 in a single cell-free reaction. ACS Synth. Biol. 1, 408–413 doi:10.1021/sb300049p 23651338

[53] Heyman Y., Buxboim A., Wolf S.G., Daube S.S. and Bar-Ziv R.H. (2012) Cell-free protein synthesis and assembly on a biochip. Nat. Nanotechnol. 7, 374–378 doi:10.1038/nnano.2012.65 22635100

[54] Eisenstein M. (2016) Living factories of the future. Nature 531, 401–403 doi:10.1038/531401a 26983542

[55] Voloshin A.M. and Swartz J.R. (2005) Efficient and scalable method for scaling up cell free protein synthesis in batch mode. Biotechnol. Bioeng. 91, 516–521 doi:10.1002/bit.20528 15937883

[56] Hartley A.D., Santos M.A.S., Colthurst D.R. and Tuite M.F. (1996) Preparation and use of yeast cell-free translation lysate In Yeast Protocols, pp. 249–258. Humana Press, New Jersey 10.1385/0-89603-319-8:249 8924986

[57] Gan R. and Jewett M.C. (2014) A combined cell-free transcription-translation system from *Saccharomyces cerevisiae* for rapid and robust protein synthesis. Biotechnol. J. 9, 641–651 doi:10.1002/biot.201300545 24677809

[58] Moore S.J., Lai H.-E., Needham H., Polizzi K.M. and Freemont P.S. (2017) *Streptomyces venezuelae* TX–TL — a next generation cell-free synthetic biology tool. Biotechnol. J. 325, 1600678 doi:10.1002/biot.201600678 28139884

[59] Thompson J., Rae S. and Cundliffe E. (1984) Coupled transcription-translation in extracts of *Streptomyces lividans*. Mol. Gen. Genet. 195, 39–43 doi:10.1007/BF00332721 6593562

[60] Jones G.H. (1975) Macromolecular synthesis in *Streptomyces antibioticus*: in vitro systems for aminoacylation and translation from young and old cells. J. Bacteriol. 124, 364–372 PMID:51847 10.1128/jb.124.1.364-372.1975 PMC235904

[61] Li J., Wang H., Kwon Y.-C. and Jewett M.C. (2017) Establishing a high yielding *Streptomyces*-based cell-free protein synthesis system. Biotechnol. Bioeng. doi:10.1002/bit.26253 28112394

[62] Kelwick R., Webb A.J., MacDonald J.T. and Freemont P.S. (2016) Development of a *Bacillus subtilis* cell-free transcription-translation system for prototyping regulatory elements. Metab. Eng. 38, 370–381 doi:10.1016/j.ymben.2016.09.008 27697563

[63] Cimermancic P., Medema M.H., Claesen J., Kurita K., Wieland Brown L.C., Mavrommatis K. et al. (2014) Insights into secondary metabolism from a global analysis of prokaryotic biosynthetic gene clusters. Cell 158, 412–421 doi:10.1016/j.cell.2014.06.034 25036635 PMC4123684

[64] Albayrak C. and Swartz J.R. (2013) Cell-free co-production of an orthogonal transfer RNA activates efficient site-specific non-natural amino acid incorporation. Nucleic Acids Res. 41, 5949–5963 doi:10.1093/nar/gkt226 23589624 PMC3675464

[65] Brustad E.M. and Arnold F.H. (2011) Optimizing non-natural protein function with directed evolution. Curr. Opin. Chem. Biol. 15, 201–210 doi:10.1016/j.cbpa.2010.11.020 21185770 PMC3080047

[66] Wang H.H., Isaacs F.J., Carr P.A., Sun Z.Z., Xu G., Forest C.R. et al. (2009) Programming cells by multiplex genome engineering and accelerated evolution. Nature 460, 894–898 doi:10.1038/nature08187 19633652 PMC4590770

[67] Wang H.H., Huang P.-Y., Xu G., Haas W., Marblestone A., Li J. et al. (2012) Multiplexed *in vivo* his-tagging of enzyme pathways for *in vitro* single-pot multienzyme catalysis. ACS Synth. Biol. 1, 43–52 doi:10.1021/sb3000029 22737598 PMC3377159

[68] Akabayov B., Akabayov S.R., Lee S.-J., Wagner G. and Richardson C.C. (2013) Impact of macromolecular crowding on DNA replication. Nat Commun. 4, 1615 doi:10.1038/ncomms2620 23511479 PMC3666333

[69] Ellis R.J. (2001) Macromolecular crowding: an important but neglected aspect of the intracellular environment. Curr. Opin. Struct. Biol. 11, 114–119 doi:10.1016/S0959-440X(00)00172-X 11179900

[70] Vendeville A., Larivière D. and Fourmentin E. (2011) An inventory of the bacterial macromolecular components and their spatial organization. FEMS Microbiol. Rev. 35, 395–414 doi:10.1111/j.1574-6976.2010.00254.x 20969605

[71] Martin W., Baross J., Kelley D. and Russell M.J. (2008) Hydrothermal vents and the origin of life. Nat. Rev. Microbiol. 6, 805–814 doi:10.1038/nrmicro1991 18820700

[72] Caschera F. and Noireaux V. (2014) Integration of biological parts toward the synthesis of a minimal cell. Curr. Opin. Chem. Biol. 22, 85–91 doi:10.1016/j.cbpa.2014.09.028 25285755

[73] Forster A.C. and Church G.M. (2006) Towards synthesis of a minimal cell. Mol. Syst. Biol. 2, 45 doi:10.1038/msb4100090 16924266 PMC1681520

[74] Li J., Gu L., Aach J. and Church G.M. (2014) Improved cell-free RNA and protein synthesis system. PLoS ONE 9, e106232 doi:10.1371/journal.pone.0106232 25180701 PMC4152126

